# Clinical characteristics, antimicrobial resistance, and risk factors for mortality in paediatric invasive pneumococcal disease in Beijing, 2012–2017

**DOI:** 10.1186/s12879-022-07179-8

**Published:** 2022-04-05

**Authors:** Man Jiang, Xi Wang, Liang Zhu, Yong-hong Yang, Kai-hu Yao, Fang Dong, Wei Shi, Qing Wang, Wen-qi Song, Gang Liu

**Affiliations:** 1 Key Laboratory of Major Diseases in Children, Ministry of Education, Department of Infectious Diseases, National Center for Children’s Health, China, Beijing Children’s Hospital Affiliated to Capital Medical University, No. 56 Nan Lishi Road, Beijing, 100045 China; 2 Department of Infectious Diseases, Shenzhen Children’s Hospital, Shenzhen, 518035 China; 3 Key Laboratory of Major Diseases in Children, Ministry of Education, National Key Discipline of Pediatrics (Capital Medical University), National Center for Children’s Health, Beijing Pediatric Research Institute, Beijing Children’s Hospital, Capital Medical University, Beijing, 100045 China; 4 Department of Laboratory Medicine, Beijing Children’s Hospital, Capital Medical University, Beijing, 100045 China

**Keywords:** Invasive pneumococcal disease, Serotype, Microbial sensitivity tests, Risk factors, Mortality

## Abstract

**Background:**

To analyse clinical characteristics, antibiotic susceptibility, and risk factors for mortality in paediatric invasive pneumococcal disease (IPD) in Beijing.

**Methods:**

Paediatric IPD patients in our hospital were retrospectively collected from 2012 to 2017. Clinical manifestations, laboratory tests, antimicrobial susceptibility and serotype of isolates, and risk factors for mortality of IPD were analysed.

**Results:**

Overall, 186 IPD cases were enrolled. The major manifestations were meningitis (76), pneumonia with bacteraemia (60), bacteraemia without focus (21), and pneumonia with empyaema (22). Of 72 cases with underlying diseases, leukaemia (18.0%), congenital heart disease (15.3%), primary immunodeficiency disease (12.5%), nephrotic syndrome (12.5%), and cerebrospinal fluid leakage (12.5%) were most common. In total 96.9% of isolates would have been covered by the pneumococcal conjugate vaccine (PCV13), including 19F (32.8%), 19A (23.4%), 4 (17.2%), and 23F (9.4%). Nonsusceptibility rates of penicillin, cefotaxime, and cefepime among nonmeningitis patients increased between 2012 and 2017; The mortality rate was 21.5%. Meningitis, respiratory failure, multiple organ failure, and white blood cell count < 4000 cells/μL were independent risk factors for mortality.

**Conclusion:**

Meningitis was the most common clinical manifestation of IPD, and was frequently associated with death. Strains in the PCV13 vaccine would cover most of the cases, and so wider use of PCV13 should be considered.

## Background

*Streptococcus pneumoniae* is a major cause of bacterial meningitis, septicaemia, and pneumonia worldwide [1] and is a commensal bacterium that colonises the pharynx and upper respiratory tract of healthy individuals. However, it can spread and establish at various normally sterile sites, such as blood, cerebrospinal fluid (CSF), and the pleural space, causing invasive pneumococcal disease (IPD) [2]. Mortality rates of children with IPD are approximately 5.3%–27.5% and can be even higher, depending on the IPD type [3–5]. In 2010, Navarro-Torne et al. [6] found that age, meningitis, non-PCV serotypes among children < 5 years of age, and penicillin nonsusceptibility were risk factors for mortality in Europe. A study from England and Wales found that infants aged < 1 year and diagnosis of meningitis contributed to half of the fatal cases in childhood [7]. Children with underlying disease have also been reported to have a higher mortality rate [8, 9]. An analysis of 134 isolates in Taiwan showed the major risk factors for IPD-related death were inappropriate initial therapy of giving ceftriaxone to patients infected by ceftriaxone-resistant strains [10]. Few data are available about the IPD-related case mortality rate and risk factors for mortality in mainland China.

The antibiotic resistance of *S. pneumoniae* is increasing in China. Between 2006 and 2008, Xue et al. [11] collected 171 strains isolated from children with IPD from 11 centres in China. Their drug sensitivity analyses revealed that 89.5% were multidrug-resistant, and penicillin resistance was observed in 76.6% of meningeal isolates. A study of children with IPD aged < 5 years in China between 2009 and 2011 showed the multidrug resistance rate was as high as 96.3% and the penicillin resistance rate was 59.26% [12]. Lyu S et al. [13] researched 187 *S. pneumoniae* strains isolated from IPD patients in Beijing in 2013 and 2014 and found that the multi-drug resistance rate was 94% and the penicillin and erythromycin resistance rates were 28.5% and 100%, respectively. It is necessary to continue monitoring the changes in antibiotic resistance of *S. pneumoniae* in China.

The aim of the present study is to analyse the clinical features of paediatric IPD, antimicrobial resistance and serotype distribution of pneumococcal strains isolated from children with IPD, and the risk factors for mortality following IPD.

## Methods

### Study cohort and definition

**Fig. 1:**
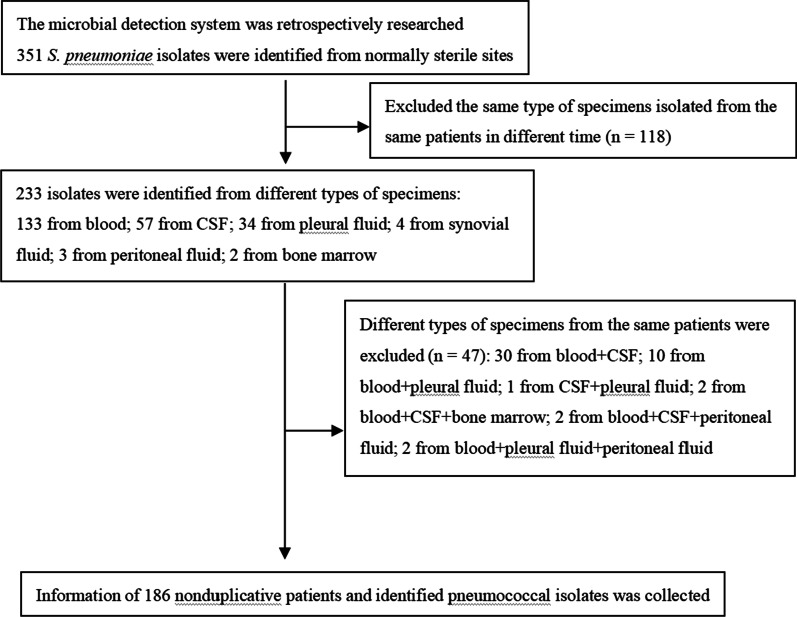
Diagram of the identification process of pneumococcal isolates

We retrospectively reviewed all invasive pneumococcal isolates between January 2012 and December 2017 in Beijing Children’s Hospital. A total of 351 *S. pneumoniae* isolates were obtained (Fig. 1). Excluding 118 repeated isolates from sampling at different times and 47 isolates from more than one sterile body site of the same patient, 186 nonduplicative patients were enrolled. For patients with multiple episodes, only the first episode was included. Clinical information was collected, including age, sex, onset season, clinical manifestations, diagnosis, underlying diseases, laboratory tests, co-infection, treatment, the types of antibiotics prescribed for IPD patients, complications, and outcome.

The diagnosis of IPD was based on clinical manifestation and cultures positive for *S. pneumoniae* that were isolated from a normally sterile body site, including blood, CSF, pleural fluid, peritoneal fluid, synovial fluid, or pericardial fluid. Nasal, ear, ocular, sputum, and bronchoscopic specimens were excluded from the study. Pneumococcal meningitis was defined as (i) identification of *S. pneumoniae* in CSF or (ii) *S. pneumoniae* cultured from blood and CSF cell counts were consistent with bacterial meningitis in combination with clinical features of meningitis. Bacteraemia without focus was defined as isolation of *S. pneumoniae* from blood associated with general symptoms without the identification of focal signs. Pneumonia with bacteraemia was defined as patients with a diagnosis of pneumonia and one or more blood cultures yielding *S. pneumoniae*. Community-acquired episode was defined by disease onset before and within 48 h after admission to the hospital. Otherwise, the episode was defined as nosocomial. The 28-day mortality rate was defined as the proportion of patients who died within 28 days after discharge. This study was approved by the regional ethical review board in Beijing Children’s Hospital, Capital Medical University (IEC-C-008-A08-V.05.1), and was carried out in accordance with the principles of the declaration of Helsinki.

### *S. pneumoniae* isolation and serotype identification

Specimens of blood, CSF, and peritoneal fluid were inoculated in 5% sheep blood agar plates, which were incubated at 35 °C in 7% CO_2_ for 18–24 h. Colonies were selected and further purified. At the same time, the optochin test was performed and the strain was identified using the VITEK 2 automatic microbiological analyser GPI card. The serotping of the *S. pneumoniae* was performed by the Quellung test using serotype specific antiserum (Statens SerumInstitut, Copenhagen, Denmark). Serotypes included by pneumococcal conjugate vaccine(PCV) were defined as PCV7 (including serotypes 4, 6B 9 V, 14, 18C, 19F, and 23F) or PCV13 (including 6 more serotypes 1, 3, 5, 6A,7F, and 19A) vaccine covered serotypes. Otherwise, non-PCV serotypes were identified. The operation steps and judgment criteria were carried out according to the literature [14].

### Antimicrobial resistance testing

Bacteria were identified by automatic bacterial identification system (VITEK 2 Compact, France) or Optochin Discs (OXOID, UK). Twelve antibiotics susceptibility (including penicillin, cefotaxime, cefepime, erythromycin, clindamycin, and tetracycline) of *S. pneumoniae* were tested by Kirby-Bauer method. When the minimum inhibitory concentration (MIC) of penicillin was ≥ 2, another E-Test method was used for supplementary experiments. The minimum inhibitory concentration (MIC) of penicillin for *S. pneumoniae* was determined in accordance with the guidelines by the Clinical and Laboratory Standards Institute (2013 edition). The American Type Culture Collection (ATCC 49,619) strain of *S. pneumoniae* was used as controls during the susceptibility test.

### Statistical analysis

Statistical analysis was performed with SPSS 17.0 for Windows. Categorical variables were presented as the number of cases and percentages and compared using the χ^2^ test or the two-tailed Fisher exact test. Continuous variables that did not follow a normal distribution were described as median with interquartile range (IQR) and compared using the Mann–Whitney *U* test. Univariable analysis was performed to identify factors associated with a fatal outcome and variables with a *P*-value of less than 0.2 in the univariate analysis were included in the final model for multivariable analysis and the odds ratio (OR) and 95% confidence interval (95% CI) were calculated. A *P*-value of < 0.05 was considered statistically significant; the Bonferroni correction was applied for multiple comparisons among different age groups.

## Results

### Demographics of children with IPD

Among the 186 patients, 111 (59.7%) were male and 75 (40.3%) were female. The median age was 20.5 months (IQR, 10.0–43.25), and 162 (87.1%) patients were aged < 5 years. There were 111 (59.7%) patients came from rural area and 75 (40.3%) patiens came from urban area. No statistical difference was observed by comparison of characteristics of subjects from the two groups, including age, sex, and clinical manifestations. Within one patient, who had completed the PCV7 series, was isolated serotype 19A. Three patients had received PCV without knowing the time to vaccination and doses. No seasonal trend was observed. The median length of hospitalisation was 19 days (IQR, 8.75–28); 62 (33.3%) patients had concurrent infection, including mycoplasma pneumoniae (16 cases), adenovirus (15 cases), influenza (13 cases), rotavirus (9 cases), Epstein–Barr virus (7 cases), respiratory syncytial virus (5 cases), and cytomegalovirus (1 case); 162 (87.1%) cases were community-acquired and 24 (12.9%) cases were nosocomial infected. Among the 24 patients with nosocomial infection, 10 (41.7%) cases were bacteremia without focus and 18 patients (75.0%) had underlying diseases, which were significantly more than that in community acquired infection group (*P* < 0.05). The serotype was identified in 6 patients with nosocomial infection, including serotype 19F and 19A( 2 cases each), 15B and 6A (1 case each). No significantly difference was found in the antibiotic resistance profiles between the nosocomial and community-acquired infection groups.

### Manifestations of IPD

Meningitis (40.9%) was the most common manifestation, followed by pneumonia with bacteraemia (32.3%) and bacteraemia without focus (11.3%) (Table 1). The distribution of manifestations varied with age. Among infants ≤ 12 months, 56.7% suffered from meningitis, compared with 25.4% among children aged 1–3 years. Pneumonia without meningitis occurred in 31.3% of infants aged ≤ 12 months and 62.7% of children aged 1–3 years.

**Table 1:** Clinical and demographic characteristics of children with invasive pneumococcal disease in different age groups•

Age (months)^※^	0–12 (*n* = 67) (%)	13–36 (*n* = 59) (%)	37–60 (*n* = 36) (%)	> 60 (*n* = 24) (%)	Total (*n* = 186) (%)	χ^2^	*P*
Sex						4.640	0.200
Male	39 (58.2)	30 (50.8)	25 (69.4)	17 (70.8)	111 (59.7)		
Female	28 (41.8)	29 (49.2)	11 (30.6)	7 (29.2)	75 (40.3)		
Disease onset season						8.446	0.490
Dec–Feb	26 (38.8)	18 (30.5)	12 (33.3)	8 (33.3)	64 (34.4)		
Mar–May	16 (23.9)	18 (30.5)	11 (30.6)	3 (12.5)	48 (25.8)		
Jun–Aug	14 (20.9)	12 (20.3)	11 (30.6)	8 (33.3)	45 (24.4)		
Sep–Nov	11 (16.4)	11 (18.6)	2 (5.6)	5 (20.8)	29 (15.6)		
Clinical manifestations							
Meningitis	38 (56.7)^a^	15 (25.4)^b^	12 (33.3)^a,b^	11 (45.8)^a,b^	76 (40.9)	13.879	0.003
-With pneumonia	24	12	4	4	44		
Pneumonia without meningitis	21 (31.3)^a^	37 (62.7)^b^	16 (44.4)^a,b^	9 (33.3)^a,b^	82 (44.6)	12.818	0.005
-with bacteraemia	18^a^	28 ^a^	10 ^a^	4^a^	60	10.130	0.017
-with empyaema	3	8	6	5	22		
-with HUS‡	0	0	2	0	2		
Bacteraemia without focus	4 (6.0)	5 (8.5)	8 (22.2)	4 (16.7)	21 (11.3)	7.002	0.064
Arthritis	4 (6.0)	1 (1.7)	0	0	5 (2.8)	2.919	0.370
-With osteomyelitis	2	1	0	0	3		
Peritonitis^†^	0	1 (1.7)	1 (2.8)	2 (8.3)	4 (2.3)		
Endocarditis	0	1	0	0	1 (0.5)		
Complications^¶^							
Neurological complications	28 (73.7)^a^	8 (53.3)^a,b^	3 (25.0)^b^	3 (27.3)^b^	42 (55.3)	13.170	0.004
Respiratory complications	13 (28.9)^a^	22 (44.9)^a^	16 (80.0)^b^	7 (53.8)^a,b^	58 (45.7)	14.969	0.002
Disease severity							
Respiratory failure	17 (25.4)	8 (13.6)	10 (27.8)	4 (16.7)	39 (21.0)	4.014	0.260
Septic shock	5 (7.5)	4 (6.8)	2 (5.6)	1 (4.2)	12 (6.5)	0.339	1.000
HLH	4 (6.0)	4 (6.8)	4 (11.1)	0	13 (7.0)	2.986	0.411
MODS	3 (4.5)	7 (11.9)	2 (5.6)	1 (4.2)	13 (7.0)	2.678	0.433
ICU admission	36 (53.7)^a^	19 (32.2)^a^	11 (30.6)^a^	9 (37.5)^a^	75 (40.3)	8.129	0.043
Intubation	26 (38.8)^a^	15 (25.4)^a,b^	3 (8.3)^b^	5 (20.8)^a,b^	49 (26.3)	11.782	0.008
CPR	8 (11.9)	1 (1.7)	1 (2.8)	1 (4.2)	11 (5.9)	5.818	0.078
Mortality	21 (31.3)	10 (16.9)	4 (11.1)	5 (20.8)	40 (21.5)	6.878	0.076

• Data are presented as the number of cases (%) for categorical variables. HUS, haemolytic uraemic syndrome; HLH, haemophagocytic lymphohistiocytosis; MODS, multiple organ dysfunction syndrome; ICU, intensive care unit; CPR, cardiopulmonary resuscitation

^※^Multiple comparisons among different age groups by using the Bonferroni method was performed for the variables of meningitis, pneumonia without meningitis, pneumonia with bacteraemia, neurological complication, and respiratory complications. Statistically significant difference (adjusted *P*-value < 0.05) was identified between ‘a’ and ‘b’ groups, but no statistically significant difference was identified between group ‘a’ and ‘a’ or ‘b’ and ‘b’

^‡^Two of the HUS patients had concurrent pneumonia with empyaema

^†^Three of the peritonitis patients had concurrent pneumonia, and one had concurrent meningitis

^¶^Incidence of neurological complication calculated in patients with meningitis only. Incidence of pneumonia complication calculated in patients with pneumonia only

### Complications of IPD

Of the 76 children with meningitis, 42 (55.3%) had neurological complications: subdural effusion (26 cases), hydrocephalus (13 cases), cerebral hernia (10 cases), intracranial haemorrhage (7 cases), ventriculitis (3 cases), extensive cerebral parenchyma involvement (1 case), and venous sinus thrombosis (1 case). Complications of pneumonia were found in 58 children: pleural effusion (38 cases), Pyopneumothorax (13 cases), atelectasis (8 cases), pneumothorax (5 cases), bronchopleural fistula (2 cases), pneumatocele and lung abscess (2 cases each), and pulmonary haemorrhage (1 case). Among patients aged ≤ 1 year, more patients with meningitis presented with neurological complications compared with other age groups, whereas more complications of pneumonia occurred in older patients (Table 1).

### Children with IPD with underlying diseases

There were 72 (38.7%) patients with underlying diseases. The main underlying diseases were leukaemia (13 cases), congenital heart disease (11 cases, including 3 with heart surgery), CSF leakage (9 cases), nephrotic syndrome (9 cases), and primary immunodeficiency disease (9 cases). Others included neuroblastoma (5 cases), RAS-associated autoimmune leukoproliferative disorder, Langerhans cell histiocytosis, mental retardation, hearing disorder (2 cases each), systemic lupus erythaematosus, oesophageal mediastinal fistula, epilepsy, hydrocephalus, asthma, bronchiolitis obliterans, bone marrow transplantation, and cochlear implant (1 case each).

**Fig. 2:**
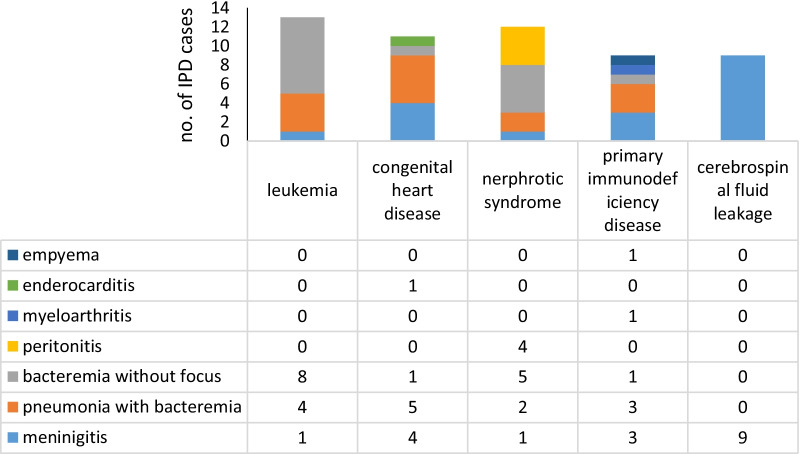
The clinical manifestations of children with invasive pneumococcal disease with underlying diseases

The major manifestations of IPD with underlying diseases were meningitis (26 cases, 36.1%), pneumonia with bacteraemia (24 cases, 33.3%), and bacteraemia without focus (17 cases, 23.6%). Peritonitis was observed in 4 (5.6%) cases, and 1 (1.4%) case was myeloarthritis. Of the 21 cases with bacteraemia without focus, 81.0% had underlying diseases, and 19% did not have underlying diseases (χ^2^ = 17.805, *P* < 0.001). It was noted that all the four patients with peritonitis had nephrotic syndrome, and three also had pneumonia with pleural effusion. All nine patients with CSF leakage presented with meningitis, and one of them had recurrent *S. pneumoniae* meningitis 4 years after the first episode (Fig. 2).

### Serotype distribution and antibiotic susceptibility results of isolates from children with IPD

**Fig. 3:**
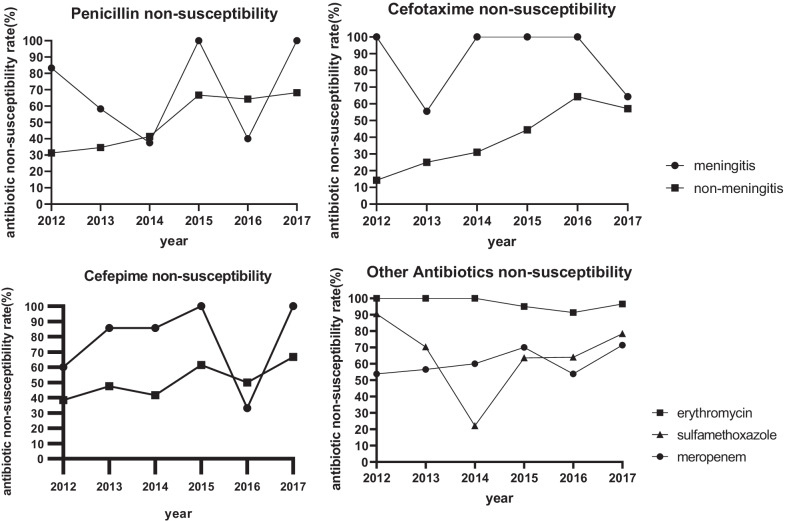
Changes in antibiotic nonsusceptibility rates (2012 to 2017) among 186 children with invasive pneumococcal disease

Antibiotic sensitivity test results were available for 181(97.3%) patients (Table 2). The number of multidrug-resistant strains (resistant to ≥ 3 antibiotics at the same time) was 147 (81.2%), 128 (87.1%) of which were resistant to erythromycin, clindamycin, and tetracycline. The nonsusceptibility rates of penicillin, cefotaxime, and cefepime among nonmeningitis patients increased from 31.3%, 14.3%, and 38.5% in 2012 to 68.2%, 57.1%, and 66.7% in 2017, respectively (Fig. 3). The nonsusceptibility rates of meningitis isolates fluctuated by year.

**Table 2:** Antibiotic susceptibility results of pneumococcal isolates from children with invasive pneumococcal disease

Antibiotic^a^	No. of isolates (%)^b^				Total no. of isolates
	Susceptible	Intermediate	Resistant	Nonsusceptible (R + I)	
Penicillin					
Meningitis^c^	16(28.1)	0	41(71.9)	41 (71.9)	57
Nonmeningitis	67 (51.9)	51 (39.5)	11 (8.5)	62 (48.1)	129
Cefotaxime					
Meningitis^c^	17 (35.4)	23 (47.9)	8 (16.7)	31 (64.6)	48
Nonmeningitis	74 (61.7)	27 (22.5)	19 (15.8)	46 (37.5)	120
Cefepime					
Meningitis^c^	10 (23.8)	12 (28.6)	20 (47.7)	32 (76.2)	42
Nonmeningitis	49 (49.5)	42 (42.4)	8 (19.0)	50 (50.1)	99
Erythromycin	4 (2.6)	0	152 (97.4)	152 (97.4)	156
Clindamycin	4 (2.6)	0	149 (97.4)	149 (97.4)	153
Tetracycline	14 (9.0)	14 (9.0)	128 (82.1)	128 (91.0)	156
SMZ-Co	47 (26.3)	22 (12.3)	110 (61.5)	110 (73.7)	179
Chloramphenicol	166 (93.3)	0	12 (6.7)	12 (6.7)	178
Levofloxacin	155 (100.0)	0	0	0 (0)	155
Meropenem	39 (39.0)	35 (35.0)	26 (26.0)	61 (61)	100
Vancomycin	181 (100.0)	0	0	0 (0)	181
Linezolid	181 (100.0)	0	0	0 (0)	181

^a^SMZ-Co, compound sulfamethoxazole

^b^Data are presented as the number of cases (%) for categorical variables

^C^For patients clinically diagnosed with meningitis and with drug sensitivity test results, the strain is derived from CSF or other sterile site samples, where priority is given to the inclusion in CSF isolates. Other sterile site samples are interpreted according to meningitis resistance criteria

**Fig. 4:**
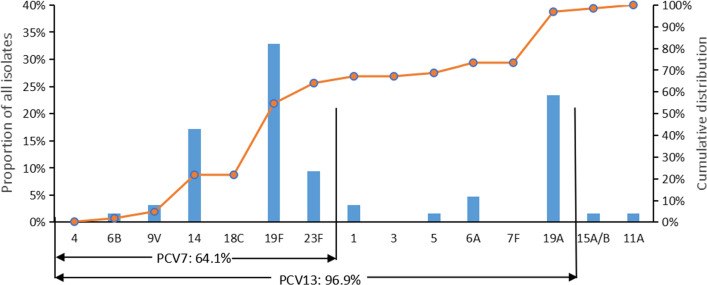
Proportionate and cumulative serotype distribution of *S. pneumoniae* isolates from IPD children

The serotype was identified in 64 patients; serotype 19F (32.8%), 19A (23.4%), and 14 (17.2%) were the most common types. PCV13 vaccine-covered serotypes accounted for 96.9% (Fig. 4).

### Antibiotic treatment

All of the 186 cases were treated with antibiotics, and 166 (89.2%) cases received more than one types of antibiotic. Before bacterial cultre, 49 (26.3%) patients had received carbapenems. After the possitive results of *S. pneumoniae* culture were reported, 11 (5.9%) cases, 100 (53.8%) cases, 66 (33.5%) cases, 69 (37.1%) cases, and 13(7.0%) cases were prescribed by penicillin, cephalosporin, meropenem, vancomycin, and macrolides, respectively. There were 87 (46.8%) patients had linezolid, 8 (4.3%) cases had teicolanin.

### Prognosis and risk factors for mortality in children with IPD

A total of 40 cases died of IPD (14 cases died in the hospital and 26 cases died of IPD within 28 days after discharge). The mortality rate was 21.5% (40/186). Among the 40 fatal cases, the median age was 12 (IQR, 5–34.25) months, 21 (52.5%) cases were aged under 1 year, and 13 (32.5%) cases had underlying diseases. There were 16 (42.1%) cases with serotype identification, which revealed that eight cases were type 19F, two cases were types 14 and 6A, respectively, and one case was type 19A, 23F, 15B, and 1, respectively. Meningitis, respiratory failure, multiple organ dysfunction syndrome, and WBC count < 4000 cells/μL were risk factors for mortality in children with IPD (Table 3).

**Table 3:** Logistic regression analysis for risk factor analysis of fatal IPD

Characteristics^a^	Outcome		Univariate analysis		Multivariate analysis^c^	
	Fatal	Nonfatal	Odds ratio (95% CI)	*P*-value	Odds ratio (95% CI)	*P*
	(*n* = 40) (%)^b^	(*n* = 146) (%)				
Age < 1 year	21 (52.5)	19 (13.0)	0.74 (0.51–1.08)	0.116		
Underlying diseases	13 (32.5)	59 (40.4)	0.71 (0.34–1.49)	0.364		
Meningitis	24 (60.0)	52 (35.6)	2.71 (1.32–5.58)	0.006	4.13 (1.65–10.38)	0.003
Septic shock	10 (25.0)	2 (1.4)	24.00 (5.00–115.17)	< 0.001		
MODS	9 (22.5)	4 (2.7)	10.31 (2.98–35.62)	< 0.001	13.53 (3.15–58.16)	< 0.001
Respiratory failure	19 (47.5)	20 (13.7)	5.70 (2.61–12.43)	< 0.001	9.00 (3.53–22.96)	< 0.001
WBC count < 4000 cells/μL	16 (40.0)	23 (15.8)	3.83 (1.75–8.37)	0.001	3.31 (1.16–9.46)	0.026
CRP > 50 mg/L	35 (87.5)	120 (82.2)	1.75 (0.57–5.38)	0.329		
Nosocomial infection	4 (10.0)	20 (13.7)	0.70 (0.23–2.18)	0.538		
Co-infection	7 (17.5)	55 (37.7)	0.35 (0.15–0.85)	0.020		
Penicillin nonsusceptibility	17 (42.5)	86 (58.9)	0.85 (0.42–1.73)	0.658		

^a^MODS, multiple organ dysfunction syndrome; WBC, white blood cell; CRP, C-reactive protein

^b^Data are presented as the number of cases (%) for categorical variables

^c^All variables with a *P*-value < 0.20 in the univariate analysis were included in the logistic regression model in the multivariate analysis. A forward stepwise selection process was utilised

## Discussion

*S. pneumoniae* is a major cause of bacterial meningitis, septicaemia, and pneumonia worldwide [1]. In the current study, Pneumococcal meningitis accounted for 40.9% of cases, being the most common manifestation of paediatric IPD, in agreement with recent reports from Shanghai (42.6%) [3], Lanzhou (34.5%) [3], and Shuzhou (31.3%) [15], China. In a study in India, the proportion of pneumococcal meningitis among children with IPD aged under 5 years between 2011 and 2015 was 35% [16]. These percentages differ from the post-PCV era; for example, a retrospective study from 2001 to 2006 in Taiwan showed the incidence of meningitis was only 8% [17]. In the period from 2006 to 2014, the incidence of meningitis in England and Wales was 22% [18]. The Active Bacterial Core Surveillance in the USA in 2017 indicated that the incidence of meningitis was 6.9%, much lower than those of pneumonia with bacteraemia (69.3%) and bacteraemia without focus (16.4%) [19].

The distribution of manifestations varied with age. More than half of the infants aged under 1 year had meningitis, in agreement with the results of a recent multicentre surveillance in China [20]. As many as 47 (25.2%) patients had more than one site of infection, which was more than reported in a previous study in China [3]. Notably, 12 cases suffered from more rare IPD manifestations, such as arthritis, peritonitis, HUS, and endocarditis, which have also been described in previous studies [21–23].

Between 11 and 44% of children with IPD have been reported to have an underlying disease [8, 24, 25]. Underlying diseases include HIV infection [8], asthma [26–28], congenital heart disease, cancer, renal diseases, and organ transplantation [29], which have been considered to be risk factors for IPD. In the present study, the underlying diseases in children with IPD showed a great variety, and the underlying disease rate was 38.7%, which is higher than in other studies in China [3, 30]. This could be explained by the fact that our hospital is a national paediatric medicine centre which houses a national haematology centre and various specialist wards to accept more children with underlying diseases. The mortality rate for children with IPD has been found to be 3 to 4 times higher if they also have an underlying disease [1, 31, 32], but we found no significant difference in mortality between children with IPD with or without underlying disease. The incidence of underlying diseases was even higher in the nonfatal group (40.4%) than in the fatal group (32.5%) (Table 3). This could be explained by several reasons. First, broad-spectrum antibiotics and more intensive therapy were given to patients with underlying diseases to effectively control the infection. Second, our hospital receives not only IPD patients from Beijing but also from around China (accounting for 73.5%) who were transferred from local hospitals because of severe conditions. Although these patients had no underlying disease, this may have induced bias of mortality. Though the underlying disease was no risk factor in the present study, we confirmed factors that were of potential concern because of the heavy burden for IPD in immunocompromised individuals.

*S. pneumoniae* drug resistance has become a global problem. The multidrug resistance rate in the present study (81.2%) was higher than that in a recent multicentre study in China (46.1%) [33] but similar to the level reported by ANSORP (83.3%), which was significantly higher than that in Asia (59.3%) [34]. The nonsusceptibility rates of penicillin, cefotaxime, and cefepime in nonmeningeal isolates increased with time. Of nonmeningitis isolates, 8.5% were resistant to penicillin, which was significantly more than the 0.7% reported by ANSORP [34] and percentages reported in other parts of China [11, 33, 35]. This discrepancy could at least partially result from the inappropriate use of antibiotics, as 49 patients (26.3%) had received carbapenems antibiotics and an even higher proportion of patients had received other types of antibiotics before bacterial culture. Therefore, continuous monitoring of penicillin-nonsusceptible *S. pneumoniae* in children with nonmeningitis IPD should be reinforced in the region.

The estimated serotype identification showed that PCV13 vaccine-covered serotypes accounted for 96.9%, similar to a recent meta-analysis in China [36, 37]. The serotype distribution suggests that, at present, PCV13 has a preventive effect on pneumococcal infection, and it is recommended to introduce PCV in China. The *S. pneumoniae* vaccine has currently not been introduced into the National Immunization Program (NIP) of China [10], but has been available by out-of pocket costs. In China, the pneumococcal vaccine is often just shorted to being referred to as the "pneumonia vaccine" [38]. The public tend to think the pneumococcal vaccine is a vaccine just against pneumonia. The real problem is meningitis caused by *S. pneumoniae*, and further information about PCV protecting against meningitis could increase its acceptance in China. Since the introduction of pneumococcal vaccines worldwide in 2000, there has been a sharp reduction in cases and deaths, including a 51% decline in infant mortality from pneumococcus between 2000 and 2015 [39]. Changes in serotype distribution have occurred in post-vaccine era [40]. Invasive pneumococcal disease incidence due to non-PCV13 serotypes doubled since the introduction of PCV7 and most of the death cases were due to nonvaccine serotypes in Englland and Wales [7, 41]. Paediatric vaccination increases the burden of non-vaccine serotype invasive pneumococcal disease in children and adults [41–43]. Reliable baseline data before vaccination are crucial to continue monitoring the occurrence of IPD after PCV in China.

The pneumococcal meningitis-associated mortality rate is close to 59% among survivors in low-income countries [16]. Large surveillance studies of IPD in Europe in 2010 [6], a Denmark cohort study in 2009 [32], and a more recent report from England and Wales [7] also showed a significant association between meningitis and death in IPD. Our analysis gave similar results. Furthermore, respiratory failure and multiple organ dysfunction syndrome were found to be independent risk factors for mortality in our study. In addition, the biomarker of WBC count < 4000 cells/μL was also associated with mortality as an independent risk factor, in agreement with previous studies [17, 44]. WBC count < 4000 cells/μL was also one of the diagnosis criteria for sepsis, which was related to poor outcomes in another study [45]. These observations suggest that early evaluation of respiratory function and signs of multiorgan dysfunction is critical to predict poor outcome. Underlying diseases [8], specific serotypes [32], and age [44] were also revealed as risk factors for mortality. Due to the limited number of cases in the present study, specific serotypes were not enrolled in the prognosis model. Moreover, we found the average age of fatal cases was lower than that of survivors, but age was no independent risk factor for mortality. Therefore, the debate continues regarding the extent to which specific serotypes, underlying diseases, and age affect the outcome of IPD, indicating further large-scale, prospective studies are warranted.

Several limitations exist in the present study. First, it was a hospital-based retrospective review. We failed to identify same prognostic factors (such as underlying diseases) in our multivariate analysis due to selection bias and the relatively small sample size. Further large-scale studies are warranted. Second, only 34.4% of the *S. pneumoniae* isolates were available for serotyping for the same reason. Serotyping of pneumococcal isolates should be encouraged in future investigations. Nevertheless, our study adds to the limited literature about IPD in China and emphasises the need for continued and improved surveillance of IPD in China.

## Conclusions

The peak age of children with IPD was < 5 years. The manifestations of IPD varied with age. The antibiotic resistance rates are of serious concern in children with IPD in China. Paediatric IPD patients with meningitis and other severe conditions, such as respiratory failure, multiple organ dysfunction syndrome, and WBC < 4000 cells/μL, should initially be considered for intensive care.

## Data Availability

The datasets analysed during the current study are available from the corresponding author on reasonable request.

## Abbreviations

IPD: Invasive pneumococcal disease
PCV: Pneumococcal conjugate vaccine
CSF: Cerebrospinal fluid
MIC: Minimum inhibitory concentration
IQR: Interqurtile range
OR: Odd ratio
HUS: Haemolytic uraemic syndrome
HLH: Haemophagocytic lymphohistiocytosis
MODS: Multiple organ dysfunction syndrome
ICU: Intensive care unit
CPR: Cardiopulmonary resuscitation
WBC: White blood cell
SMZ-Co: Compound sulfamethoxazole
